# Human and mouse TLR2 results in different activation of p38 and JNK signal pathway in HaCaT infected by *Trichophyton rubrum* and *Microsporum canis*

**DOI:** 10.3389/fimmu.2022.1063443

**Published:** 2023-01-12

**Authors:** Zhen Su, Weiwei Deng, Shuting Zhan, Meirong Li, Songchao Yin, Jian Chen

**Affiliations:** Department of Dermatology and Venerology, The Third Affiliated Hospital of Sun Yat-Sen University, Guangzhou, China

**Keywords:** TLR2, p38, JNK, *Trichophyton rubrum (T. rubrum)*, *Microsporum canis (M. canis)*

## Abstract

**Introduction:**

It has long been recognized that inflammation to dermatophyte infection is different among various hosts, but the mechanism underlying is still not well understood. Toll-like receptor (TLR2), mediates the innate immune response against dermatophyte infection and is very important to trigger the inflammatory response to dermatophytes. Considering the different amino acid sequences and structures of TLR2, we speculated that TLR2 from different hosts will activate the downstream signal pathways to varying degrees, resulting in different inflammatory responses to dermatophytes.

**Methods:**

In this study, we constructed the mice-human fusion TLR2 expressed HaCaT (mhTLR2-HaCaT) by replacing the extracellular ligand recognition region of human TLR2 with that of the mouse. Then hTLR2-HaCaT cells and mhTLR2-HaCaT cells were infected with T. rubrum and M. canis for 24 h followed by immunoblotting to asses associated proteins of p38 and JNK signal pathway.

**Results:**

Compared with that of human TLR2 expressed HaCaT (hTLR2-HaCaT), levels of phosphorylated p38 protein were increased in mhTLR2-HaCaT cells stimulated by T. rubrum for 24 h, and levels of phosphorylatedJNK and c-Jun protein were increased in mhTLR2-HaCaT cells whenstimulated with M. canis for 24 h.

**Discussion:**

Compared with hTLR2-HaCaT cells, p38 and JNK signal pathwayswere activated in mhTLR2-HaCaT after being infected by Trichophyton rubrumand Microsporum canis, respectively. Since p38 and JNK are the mainpathways that transduce the signal for host recognition of dermatophytes andmediate the downstream inflammatory response, it suggested that theinterspecific difference of TLR2 ectodomain may be one of the reasons for thedifferent inflammatory manifestations between humans and mice infected bythese two dermatophytes. Quite especially, the mouse-derived TLR2extracellular recognition region is more effective in recognizing T. rubrum andM. canis to activate the downstream signal pathways, resulting in a tenserinflammatory response against these two dermatophytes.

## 1 Introduction

Dermatophytosis is an unsolved worldwide public health problem. In immunocompetent people, it usually results in superficial infections and is not fatal. However, it is easy to relapse and reinfection, resulting in a huge economic burden and harm to patients’ quality of life. In 2017, the United States spent more than $800 million on the treatment of human dermatophytosis (1).

The inflammatory response to a certain dermatophyte infection varies from host to host. Humans are generally susceptible to anthropophilic dermatophytes, such as *Trichophyton rubrum*. The infection always accompanies by mild inflammation and tends to be chronic, easy to relapse, and unable to heal itself (2). While mice, the most commonly used experimental animals, are resistant to many species of dermatophytes, including *Trichophyton rubrum, Trichophyton mentagrophytes*, and *Microsporum canis*. Even if the experimental infectious model is constructed under certain conditions, the infection always results in severe inflammation and the pathogens were eliminated about a month after inoculation (3–5).

It has long been recognized that inflammation to dermatophyte infection is different among various hosts. While the mechanism underlying is still not well understood. A recent study has shown that even mice with combined T and B lymphocyte defects can eliminate the dermatophytes spontaneously (3). This suggested that innate immunity plays an important role in the host’s inflammatory response and outcome to dermatophyte infection.

Toll-like receptor 2 (TLR2), a single transmembrane pattern recognition receptor, consists of an extracellular domain at the N terminal, a transmembrane domain, and a conserved TIR domain at the C terminal. The extracellular domain mediates the recognition of specific pathogen-associated molecular patterns (PAMPs). After recognition of its ligand, lipopeptide, TLR2 activates mitogen-activated protein kinase (MAPK) comprising p38, JNK, ERK, and the nuclear factor kappa-light-chain-enhancer of activated B cells (NF-κB) signaling pathway through Myd88-dependent pathway (6, 7), followed by regulating the expression of cytokines and chemokines (8). It plays an important role in phagocytosis and pro-inflammatory responses against dermatophyte infection (9, 10).

Although TLR2 is a conserved protein, there are still some differences in its sequences and spatial conformations between humans and mice, especially in its extracellular ligand recognition region (11). Therefore, we speculate that the interspecific difference of TLR2 ectodomain resulted in the different inflammatory manifestations between humans and mice infected by dermatophytes. To verify this hypothesis, we replaced the extracellular domain of human TLR2 with that of the mouse by infusion clone to construct a mouse-human fusion TLR2 expression vector, and then re-expressed it into a TLR2 knockout HaCaT strain to construct mice-human fusion TLR2 expressed HaCaT (mhTLR2-HaCaT). Meanwhile, the human TLR2 vector was re-expressed into the TLR2 knockout HaCaT to construct human TLR2 re-expressed HaCaT (hTLR2-HaCaT). *Trichophyton rubrum* and *Microsporum canis* were chosen as the subjects of our study since *Trichophyton rubrum* is a representative anthropophilic dermatophyte and is the most predominant causative agent of human dermatophytosis. And *Microsporum canis* is a representative zoophilic dermatophyte and is the most predominant causative agent of dermatophytosis in domesticated pets such as dogs and cats. Then hTLR2-HaCaT and mhTLR2-HaCaT were co-cultured with *Trichophyton rubrum* and *Microsporum canis* respectively to explore the interspecies differences of TLR2 in recognition and inflammatory response to these two dermatophytes.

## 2 Material and methods

### 2.1 Dermatophytes culture and conidia collection

*T. rubrum* came from the China General Microbiological Culture Collection Center, and M. *canis* was isolated from clinical patients. These two strains were already identified based on morphological characteristics and sequencing of the internal transcribed spacer (ITS) region and D1-D2 domain of the ribosomal DNA. *T. rubrum* was cultured at 30°C on Sabouraud dextrose agar (SDA) media (12), whereas *M*. *canis* was grown at 30°C in rice medium (3g rice, 10mL double-distilled water, autoclaved 20 min at 121°C) (13). After 14 days, the conidia were harvested with 4 mL of PBS, filtered with Whatman Grade 1 qualitative filter paper, and washed twice to remove the mycelia. The collected conidia were incubated in YEPD overnight at 30°C in the thermostatic water bath.

### 2.2 Cells culture

Human immortalized keratinocytes HaCaT cell lines and JB6 mouse epidermal cell lines were purchased from iCell Bioscience Inc, Shanghai, and 293FT cells were obtained from Chen Xue-biotech company, Guangzhou. TLR2 knockout HaCaT cells were constructed in our lab using CRISPR-Cas 9 technology as described (14). The first three cells were detached by trypsin and passed once every 2 or 3 days while the last one passed the next day. All the cells were cultured in DMEM (high glucose) supplemented with 10% FBS and 1% penicillin/streptomycin.

### 2.3 Amplification of target segments of TLR2

The cDNA library of human HaCaT cells and mouse JB6 cells were generated using PrimeScript™ II 1st Strand cDNA Synthesis Kit, respectively. Subsequently, the amplification of target segments of TLR2 was performed *via* Phusion high-fidelity DNA polymerase and then obtained the signal peptide, transmembrane plus intracellular segment of human TLR2 (hTLR2), and extracellular ligand recognition region of mouse TLR2 (mTLR2). The sequences of the primers used were as follows in **Table 1**.

**Table 1:** The sequences of primes used to amplify the target segments of TLR2.

Primers	Sequences	Purpose
signal-F	ACCTCCATAGAAGATTCTAGAGCCACCATGCCACATACTTTGTGGATGGTGT	To amplify signal peptide of hTLR2
signal-R	TCACATGACAGAGACTCCTGTTCCTTGGAGAGGCTGATGATGACC	
mTLR2-F	CAGGAGTCTCTGTCATGTGATGCTT	To amplify the extracellular ligand recognition region of mTLR2
mTLR2-R	CACATGCCAGACACCAGTGCCTGGTGACATTCCAAGACGGAGGGC	
hTLR2-F	GCACTGGTGTCTGGCATGTGCTGTG	To amplify the transmembrane plus intracellular segment of hTLR2
hTLR2-R	GATCCTTGCGGCCGCGGATCCCTAGGACTTTATCGCAGCTCTCAGA	

### 2.4 In-fusion seamless cloning reaction

**Figure 1:**
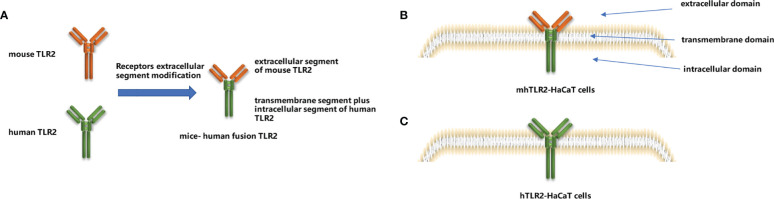
Construction of mice-human fusion TLR2 diagram **(A)**, diagram of mhTLR2-HaCaT cells **(B)** and hTLR2-HaCaT cells **(C)**.

According to the instruction of the In-Fusion HD Cloning Kit, the signal peptides of human TLR2, the extracellular segment of mouse TLR2, and the transmembrane plus intracellular segment of human TLR2 were sequentially inserted into the XbaI and EcoRI sites of the plasmid vector pLVX-Puro *via* seamless cloning, that is to say, constructing the pLVX-mhTLR2-Puro vector by replacing the extracellular ligand recognition region of human TLR2 with that of mice as illustrated in **Figure 1A**. The pLVX-hTLR2-Puro vector was also generated with full-length human TLR2 and pLVX-Puro plasmid.

### 2.5 Plasmids transformed and amplification

These two plasmids above were transformed into Trans5α E. coli competent cells and amplificated in liquid LB medium without ampicillin (Amp). After 1 h of amplification culture, the bacterial broth was coated onto solid LB plates and incubated overnight, and then single clones were picked for sequencing. Then the plasmids verified by sequencing were amplificated and extracted using the NucleoBond Xtra Midi EF kit.

### 2.6 Viruses packaging and target cells transfection

The viruses were packaged by transfection of 293FT cells based on a triple plasmid system (15). 293FT cells were seeded into six-well plates at a density of 3×10^5^ per well. After 24 h, transfection was performed using lipofectamine 2000 according to the instruction. Two packaging plasmids, pSPAX2 (1500 ng) and pMD2 (500 ng), and the target vector, pLVX-mhTLR2-Puro vector (4500 ng) were co-transfected per well. Approximately 2 mL of virus supernatants were collected at 24 h and 48 h, respectively, then the supernatants were filtered through a 0.45 µM filter and infected TLR2 knockout HaCaT cells which were seeded into six-well plates at 3×10^5^ per well and cultured for 24 h. After 48 h of infection, the cells were screened by puromycin (final concentration of 1 µg/µL) and then verified by immunoblotting. After completing the above process, mhTLR2-HaCaT cells were successfully constructed as depicted in **Figure 1B**. Construct hTLR2-HaCaT cells by replacing the target plasmid with a pLVX-hTLR2-Puro vector and proceed as above, which was illustrated in **Figure 1C**.

### 2.7 Infection of HaCaT cells by dermatophytes

The mhTLR2-HaCaT cells and hTLR2-HaCaT cells were digested with trypsin and inoculated in six-well plates at a density of 5×10^5^ per well, and the medium was changed after 24 h. Conidia of *T. rubrum* and *M. canis* were added at a density of MOI=1 for co-culture for 24 h.

### 2.8 Immunoblotting

After co-culture for 24 h, total proteins were extracted after lysis of re-expressed cells with RIPA lysate. After electrophoresis by 10% SDS-PAGE with 15 µg per well, proteins were transferred onto the PVDF membrane. Next, the members were blocked with 5% bovine serum albumin (BSA) and incubated with antibodies against c-Jun (60A8, CST), JNK(EPR16797-211, Abcam), p38 (D13E1, CST), and their phosphorylated proteins, phospho-c-Jun (Ser63) (54B3, CST), phospho-c-Jun (Ser73) (D47G9, CST), phospho-SAPK/JNK (Thr183/Tyr185) (98F2, CST), Phospho-p38 MAPK (Thr180/Tyr182) (D3F9, CST), human TLR2(D7G9Z, CST), mouse TLR2(ab209217, Abcam), and GAPDH protein(GB11002, Servicebio), respectively, overnight at 4°C, as GAPDH was an internal reference control. A secondary antibody was incubated at room temperature for 1h and then chemiluminescence developed. All the primary antibodies were used at 1:1000 dilution and secondary antibodies at 1:5000.

## 3 Results

### 3.1 Immunoblotting validation of hTLR2-HaCaT cells and mhTLR2-HaCaT cells

**Figure 2:**
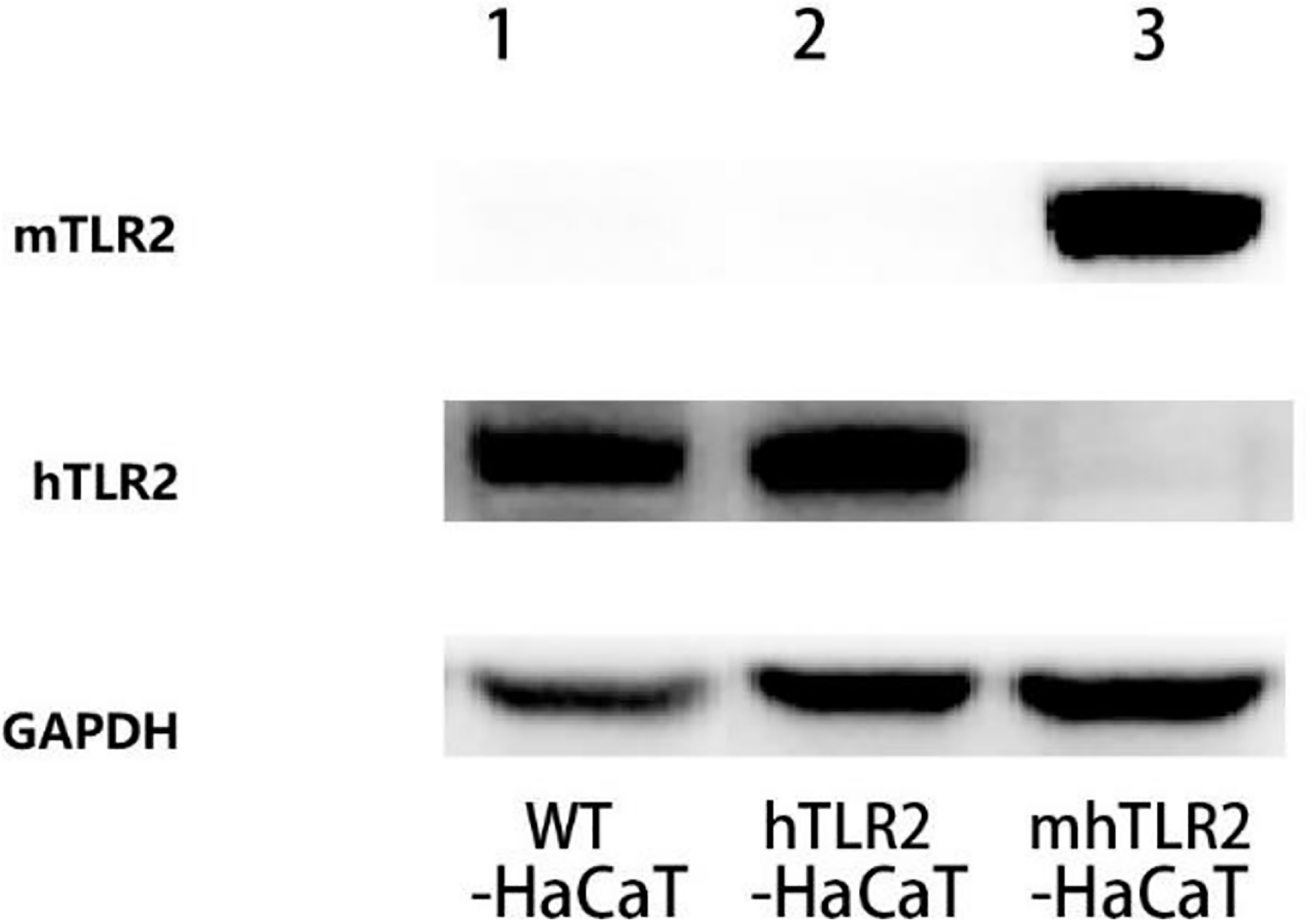
The immunoblotting results of TLR2 protein. Line 1 for WT-HaCaT cells, line 2 for hTLR2-HaCaT cells, and line 3 for mhTLR2-HaCaT cells. Representative data are presented from one of the three independent experiments. (mTLR2 represents mouse TLR2; hTLR2 represents human TLR2).

As shown in **Figure 2**, the hTLR2-HaCaT cells expressed only human TLR2 and not mouse TLR2 by immunoblotting, while the mhTLR2-HaCaT cells expressed only mouse TLR2 and not human TLR2. It was demonstrated that the two strains of cells had been successfully constructed.

### 3.2 p38 and JNK signal pathway in hTLR2-HaCaT cells and mhTLR2-HaCaT cells infected with *T. rubrum* and *M. canis*

**Figure 3:**
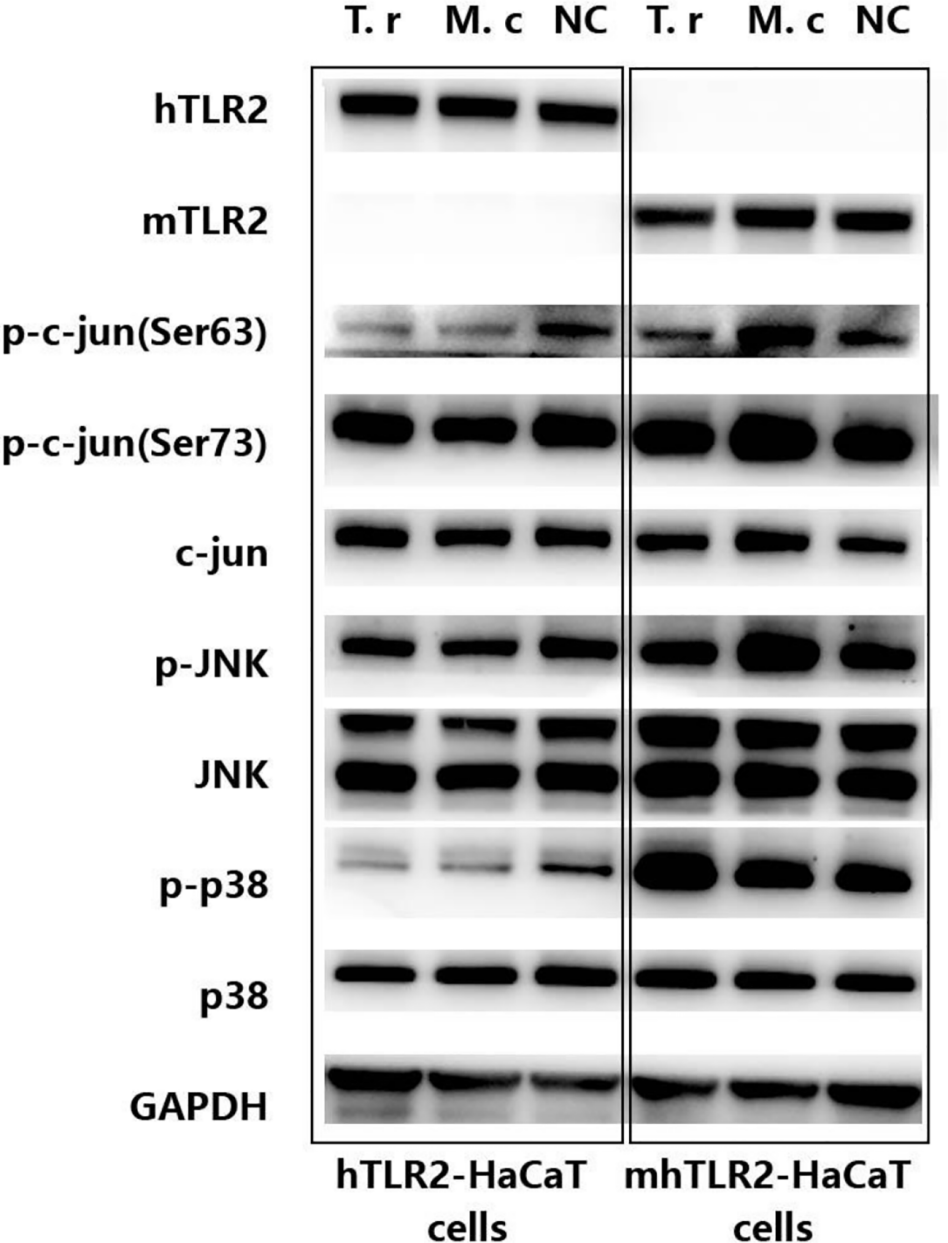
Immunoblotting results of proteins from c-Jun and p38 signaling pathway in hTLR2-HaCaT cells, mhTLR2-HaCaT cells co-cultured with *T. rubrum* and *M. canis* for 24 h. Representative data came from one of two independent experiments. (T. r represents *T. rubrum*; M. c represents *M. canis*; NC represents negative control).

As shown in **Figure 3**, compared with hTLR2-HaCaT cells, mhTLR2-HaCaT cells expressed higher levels of phosphorylated p38 protein when stimulated by *T. rubrum* for 24 h and expressed higher levels of phosphorylated JNK and c-Jun protein after stimulation with *M. canis* for 24 h.

## 4 Discussion

Generally, the dermatophytes were confined in the epidermis. As the constructed cells of the epidermis, keratinocytes can recognize and perceive PAMPs of invasive dermatophytes through pattern recognition receptors on their surface, such as TLR2, and trigger the inflammatory responses by activating downstream signaling pathways (10, 12, 16, 17). HaCaT cell, the immortalized keratinocytes cell line, is the well-established research model of human keratinocytes to simulate dermatophyte infection (10, 16, 18, 19). Therefore, HaCaT was used to carry out our study.

Innate immune response to fungi is mainly mediated by MAPK and NF-κB signaling pathways (20–22). While, different pathogenic fungi or even the different morphological forms of fungi will activate their preferential pathways to a certain degree (19, 21). As for dermatophyte infection, it seems that the MAPK pathway, rather than the NF-κB pathway, plays a more important role in antifungal immunity (14). By the global gene expression analysis, we found that *c-Jun* was the core gene when the HaCaT was challenged with *M. gypseum*. Further immunoblotting showed that Jun N-terminal protein kinase (JNK) and p38 pathways were significantly activated. While these pathways were largely unchanged when the HaCaT was challenged with the anthropophilic *T. rubrum* (19). Achterma et al. (12), combined with primary keratinocytes and organotypic epidermal models also found that two branches of the MAPK signaling pathway, p38, and JNK, were activated by *Trichophyton equinum*. Afterward, the cascade activation of the MAPK pathway leads to the secretion of IL-1α, GM-CSF, and IL-8. While, the NF-κB pathway was largely unchanged and the other major MAPK pathway, extracellular signal-regulated kinases 1 and 2 (ERK1/2), was even inhabited.

In the present study, we found that after being infected by *Trichophyton rubrum*, the p38 signal pathway was activated in mhTLR2-HaCaT cells, and the JNK/c-Jun signal pathway was significantly activated after *Microsporum canis* infection, while these two pathways were unactivated when hTLR2-HaCaT cells were challenged by each of the two dermatophytes. This indicated that p38 and JNK pathways in human keratinocytes against these two dermatophytes are active after replacing the extracellular domain of human TLR2 with that of mouse TLR2. The most reasonable explanation for this phenomenon is that the spatial structure of the extracellular ligand recognition region of mouse TLR2 is more prone to recognize the PAMPs of these two dermatophytes to activate the downstream signal pathways.

Jin et al. (11), found that although the backbone structures of the human and mouse TLR2s are practically identical, their side chains in the lipid-binding pocket differ significantly in sequences and structures. The sequence differences, including Thr335 to Leucine, Pro306 to Leucine, and Leu266 to Phenylalanine cause the shape of the lipid-binding pocket to differ substantially between mouse and human TLR2s. This resulted in more efficient recognition and binding of lipopeptides with shorter lipid chains by mouse TLR2 than human TLR2. So, it implies that the PAMPs of these two dermatophytes may contain short lipid chain lipopeptides, which were prone to be recognized by the mouse TLR2, not the human TLR2.

By the way, our results also show that different dermatophytes have their preferred signaling pathways, the infection of *Trichophyton rubrum* is more likely to cause the activation of the p38 pathway, while the infection of *Microsporum canis* is more likely to activate the JNK/c-Jun pathway. This is likely to be one of the mechanisms by which the same host produces different inflammatory responses after infection with different dermatophytes.

## Data availability statement

The original contributions presented in the study are included in the article/Supplementary Material. Further inquiries can be directed to the corresponding author.

## Author contributions

JC, conceptualization, funding acquisition, and writing –original draft; ZS, methodology and writing – review & editing; WD and SZ, resources and methodology; ML and SY; resources. All authors contributed to the article and approved the submitted version.
